# Spike timing jitter is beneficial in neural spike coding - A case of the mammalian MSO sound localization circuit

**DOI:** 10.1186/1471-2202-12-S1-P193

**Published:** 2011-07-18

**Authors:** Petr Marsalek

**Affiliations:** 1 Institute of Pathological Physiology, First Medical Faculty, Charles University of Prague, Czech Republic

## 

Auditory brainstem calculates horizontal direction (azimuth) of sound from the ITD (interaural time delay). Jeffress proposed in 1948 that sound source azimuth is computed by a neural circuit consisting of an array of delay lines [1]. This is the case in birds, but probably not in mammals [2]. We studied how the ITD-s (shorter than typical action potential duration) are processed within a stochastic model of the MSO (medial superior olive), mammalian azimuth decoder circuit [3]. The MSO circuit makes use of its intrinsic noise, yet spike timing precision in the circuit is essential. Therefore a classification of dynamical observables is needed. Most of them can be described as random variables. Introducing noisy, from trial to trial non-repetitive stimulation in binaural cochlear implants sometimes leads to a better performance [4]. Part of it can be attributed to stochastic processing.

We present our current results as a case study showing multitude of roles of (random versus deterministic) spike timing in a neural circuit. In particular, either phenomenological (excitatory-excitatory and excitatory-inhibitory units), or electrophysiological (excitatory and inhibitory synapses) classification of individual elements of the circuit might be abstracted in an explanation stressing the neural computation in the circuit. We study various parameters of the MSO model and their influence on the output of the model. Finally we evaluate the performance of the model using the concept of ideal observer.
